# Editorial Comment: Laser therapy for urinary incontinence and pelvic organ prolapse: a systematic review

**DOI:** 10.1590/S1677-5538.IBJU.2021.03.04

**Published:** 2021-03-25

**Authors:** Cássio L. Z. Riccetto

**Affiliations:** 1 Universidade Estadual de Campinas Faculdade de Ciências Médicas Divisão de Urologia Feminina CampinasSP Brasil Divisão de Urologia Feminina - Faculdade de Ciências Médicas da Universidade Estadual de Campinas – UNICAMP, Campinas, SP, Brasil.

K Mackova^1^^,^^2^, L Van Daele^3^, A-S Page^4^, I Geraerts^5^^,^^6^, L Krofta^2^, J Deprest^1^^,^^4^

BJOG. 2020 Oct;127(11):1338-1346.

## COMMENT

This paper's objective was to present a systematic review on the use of LASER therapy for the treatment of urinary incontinence (UI) and pelvic organ prolapse (POP). Erbium (ER-YAG) and carbon dioxide (CO2) LASERs were introduced as a new non-invasive treatment in gynecology, initially for the so-called menopausal genitourinary syndrome, and at a later date also being considered as a treatment option for UI and mild to moderate POP. The authors selected 31 studies involving 1530 patients in accordance with the PRISMA-guidelines including either ER-YAG (n = 21), CO2 (n = 9) LASERs, or both. A single RCT was included, in which the turned off laser probe was applied to the vagina, as sham group (1), and two studies were controlled cohort series.

All studies reported significant improvement both for objective and validated subjective IU outcome measures. Three studies addressed urge incontinence as a primary endpoint. POP was a primary endpoint in a single study (2), which reported anatomical improvements to grade 0 or 1 in 85% of the subjects and to grade 2 in 15% after two to five LASER therapy sessions. Lower cystocele grades were associated with a higher success rate. The heterogeneity of the data did not allow for the development of a meta-analysis. The authors concluded that although short term results of LASER therapy for UI and POP seem beneficial, current information on long term results, cost-effectiveness compared to well-established surgical treatments and even about which LASER model to use are lacking.

This systematic review stands out due to its high scientific standards and by being the first to include the controversial topic of LASER use in POP treatment. Be that as it may, we must highlight that the quality of the majority of the studies included is still poor, both in terms of sample sizes and also follow-up. In addition, as the physical characteristics of ER-YAG and CO2 LASERs are quite different, conclusions on the effectiveness and adverse effects of these therapies is still very limited.
